# Multilateral Development Banks as Agents of Connectivity: the Asian Development Bank (ADB) and the Asian Infrastructure Investment Bank (AIIB)

**DOI:** 10.1007/s12140-023-09408-6

**Published:** 2023-05-22

**Authors:** Katja Creutz

**Affiliations:** Finnish Institute of International Affairs/Global Security and Governance Research Programme, Helsinki, Finland

**Keywords:** Connectivity, Asia, China, Japan, Asian Development Bank, Asian Infrastructure Investment Bank

## Abstract

The article explores connectivity from the prism of multilateral development banks. It recognises that theorising on connectivity is increasing, but as connectivity agency is less addressed, it aims to contribute to discussions on who the agents of connectivity are and how they contribute thereto. By analysing the Asian Development Bank (ADB) and the Asian Infrastructure Bank (AIIB) respectively, the article sets forth a three-dimensional agency: strategic, developmental and financial. This is in line with the multi-layered nature of the banks, which reflects the broader member states and key state interests. Next to their role as banks, they contribute to the understanding of development, in addition to which they represent key shareholder interests. The banks are both to be seen as nodes of connectivity and as active agents in realising connectivity for others.

## Introduction

Connectivity, which is to be understood as ‘all the ways in which states, organisations (commercial or else) and societies are connected to each other and interact across the globe’ [75:1], has won ground as an analytical framework for how to understand the development of international relations in the last decade. This ‘latest global buzzword’ of our times [46, 43: 299] is pursued and developed by a broad range of actors, such as China and Asian states more broadly, but also sui generis bodies like the European Union (EU), and international institutions like the Association of South East Asian Nations (ASEAN). While more and more research is focussing on developing connectivity theory from different angles ranging from logics to spheres [44, 71], connectivity agency per se is rarely analysed, particularly outside the realm of states. This article posits that it is relevant to ponder who the agents of connectivity are, what role they play and how they connect to larger connectivity ideas.

While states appear central to connectivity, they rely both indirectly and directly upon a broad range of other actors to materialise and deepen connectivity. Fora for international dialogue, businesses and multilateral development banks are mentioned as connectivity agents, thus intersecting the public and private spheres. While non-state actors, such as corporations, are considered vital to the construction of so-called hard connectivity [75], little is said about multilateral development banks (MDBs) despite the fact that they engage in development and infrastructure lending and at times seek expressly to increase connectivity. As financial actors of connectivity, the property and mission of MDBs require further examination, which justifies scholarly interest in the performance of specific banks. This article aims to discuss connectivity agency from the prism of multilateral development banks, and through two case studies in particular: the Asian Development Bank (ADB) and the Asian Infrastructure Investment Bank (AIIB). The focus will be on Asian development banks, partly because the estimated need for infrastructure investment in Asia is enormous exceeding USD 20 trillion by 2030 [68: 690]; partly for the reason that connectivity has a particular Asian element to it as it is spearheaded by China and Japan [69]. In its discussion on connectivity agency, this article will draw on the conceptual underpinnings of connectivity raised by Gaens, Sinkkonen and Vogt [44], claiming that there are dimensions of connectivity (agency) that ‘deserve special emphasis’ [44]. These include the following: (1) intentionality or active agency, (2) perpetual change and (3) ‘imagined futures’ [31, 44]. The main scholarly contribution of the article lies in complementing existing strategies or theories of connectivity as they often fail to sufficiently ponder the connectivity agency of non-state actors. Moreover, academic literature on MDBs and connectivity is rather limited.

The article will focus on the following research questions: what kind of actors are multilateral development banks in infrastructure connectivity and how does this play out in the ADB and AIIB? How is their agency constructed, and how do connectivity underpinnings raised by Gaens, Sinkkonen and Vogt take shape within MDBs? In the pursuit of this mission, the article will use materials from the fields of international relations, international law and international political economy, while benefitting from the banks’ foundational instruments, official policies and portfolio. The article will start with a general outline of the concept of connectivity, connectivity agency and MDBs. It analyses the twofold rationale of MDBs in general together with the multilayered agency that the banks epitomise (“Connectivity, Connectivity Agency and Multilateral Development Banks”). From the discussion of the nature of the MDB agency, the study proceeds to the two case studies of the ADB and AIIB respectively (“The Asian Development Bank and Connectivity” and “The Asian Infrastructure Investment Bank and Connectivity”). It will explore the formal and informal role played by connectivity for the operation of the banks, as well as consider the two-way relationship with other connectivity actors, most notably states. The main analytical part is formed by the discussion on the MDB connectivity agency drawing from lessons learned in the case studies as well as relevant literature (“Connectivity and the Three-Dimensional Agency of MDBs: Strategic, Developmental, and Financial”). The article adapts the conceptual underpinnings of Gaens, Sinkkonen and Vogt [44] to the specific case of MDBs, arguing that in terms of connectivity, their agency is three-dimensional: strategic, developmental and financial. While the first role stresses MDBs as objects of being connected, the second and third ones understand them to be subjects, that is ‘connectors’. Although all three different roles that MDBs play are linked and support each other, tensions between them may also arise.

## Connectivity, Connectivity Agency and Multilateral Development Banks

The starting point of this article is the empirical reality that MDBs are relevant for connectivity and hence one actor among many that contribute to the phenomenon. The forging of relations within or between societies through various ties is not a natural, self-driven phenomenon but is produced [44]. The rise of the concept of connectivity, and concomitantly part of the political economy within which the MDBs act, has been linked to the broader transformations of the international order, in which the unipolar system and American hegemony are being replaced by nascent multipolar ordering [59]. This incremental change in the international order has brought along alternative institution-building by China in the field of international finance governance, as exemplified by the AIIB. The rise of China, and Asia more broadly, has also brought different connectivity strategies to the forefront without the emergence of one single understanding of the term. In particular, infrastructure connectivity has become a standalone concept as it plays a significant role in broader connectivity discourses [69], and it also features as a specific connectivity sphere in efforts to theorise connectivity [44]. In this article, infrastructure connectivity will be understood as a broad concept that encompasses traditional sectors such as road, transport, water and sanitation and energy, but also newer or softer forms of connectivity pertaining to, for example, the digital domain or trade policies. It is also noteworthy that the MDBs themselves state that one project may at the same time ‘address multiple operational priorities’ [11: 15], as (infrastructure) connectivity projects may also feed into tackling, for example, climate change, or vice versa. Yet, only a part of what the MDBs finance can be categorised as connectivity. For example, while building telecommunications infrastructure can be called infrastructure connectivity, projects focussing on efficiency improvement generally cannot.

### State-centredness of Connectivity

Conceptualisations of connectivity often identify states as the central actors being largely silent on other actors or simply mentioning them in passing [2: 2]. To be sure, states, or aggregations of states in one way or another, form the essential actors of the politics of connectivity. China features essentially in all analyses of connectivity, as it is a significant actor both in geopolitical and geoeconomic terms regionally and globally [86]. Its numerous connectivity initiatives—with the Belt and Road Initiative (BRI) at the forefront [86]—have defined much of the connectivity discourse to which other states or organisations have responded. Connectivity strategies of their own have been developed by Japan and the EU, but also India, South Korea and ASEAN, as well as Russia in Central Asia to a lesser degree [38]. What is often neglected in connectivity ‘theorising’, however, is the intra-state level; in practical terms, connectivity is often a local matter, which is ignored by the state-level emphasis on connectivity [71: 2274]. State-driven competitive connectivity often overshadows subnational initiatives to build connections, thereby ‘silenc[ing] subnational voices in the periphery’ [71: 2273].

The strong emphasis on state-driven connectivity can be seen as surprising against the backdrop of connectivity’s basis in globalisation theory as well as the nearly all-encompassing usage of the term [71: 2269]. Indeed, as put by Anthony and others, ‘[c]onnectivity is not the exclusive domain of any one actor’ [2: 5]. Naturally, some analyses of the agency of non-state actors exist, but they tend to be more limited in scope and less theorised. Yet, connectivity is a term employed by a variety of economic and political actors, including international and regional organisations or more loose cooperation formats, such as G20, ASEAN or the Asia-Europe meeting (ASEM) [2: 5]. The role of the private sector is also significant for connectivity as the concept is based on the flow of goods, services, information and people. Especially the intertwined actions of states and corporations are seen to be integral in connectivity, where the latter seeks to maximise profit, and the former, among other things, mixes up ‘economic agendas with geopolitical goals’ [42]. Also, societies are considered actors embedded in connectivity [75].

Multilateral development banks are mentioned among the actors of connectivity [44], as there are multiple and multidirectional links between connectivity and development lending as performed by MDBs. First, multinational development banks are actors in economic and social development by virtue of providing financial assistance to developing states [35: 1]. Development lending, in particular infrastructure lending, is an acknowledged tool to build connections [60: 21]. MDBs, such as the AIIB and the New Development Bank (NDB), are themselves considered to be embodiments of connectivity [71: 2268]. Second, connectivity as a strategy also instructs the activities of the banks, especially those MDBs that are involved in infrastucture. For example, the World Bank explains it to be ‘a leader in connectivity and logistics performance evaluation’ when it comes to trade facilitation [84]. The MDBs are also knowledge-producers concerning different aspects of connectivity, whether relating to trends in economic connectivity or identifying the need for connectivity in different regions. Yet, in terms of agency, the MDBs are special as they are neither state actors nor clear-cut non-state actors. Therefore, intentionality, which according to Gaens, Sinkkonen and Vogt is a crucial feature of connectivity [44], becomes difficult to construct and grasp.

### The Nature of Multilateral Development Bank Agency

Having established that different connectivity theories or strategies rarely address agency in connectivity as such, it is pertinent to address the question: what kind of actors are multilateral development banks and what are their links to connectivity? This section will address the structural context within which the agency of the MDBs is realised. To start with, multilateral development banks are regional or international organisations that ‘arrange loans, credits, and guarantees for investments in countries, generally with the aim of fostering economic growth’ [29: i]. The MDBs mostly lend to states, although lending to the private sector is growing [37: 114]. As indicated by the name, their main characteristics are being multilateral,^1^ that is, jointly constituted by a number of states, focussing on development through an economic method, and being banks that provide loans, not grants [29]. They have a constitutive agreement and they are organised as ‘shareholding corporations, with the size of their capital bases determining their lending capacity’ [29: 2]. It is noticeable that the shareholders are almost exclusively states [37: 115]. The World Bank is the archetype MDB, which the other banks have remodelled [57]. Today, there are around thirty MDBs, the newest one being the AIIB. They work in a range of different sectors, although infrastructure funding used to be at the core of their financing [29]. MDBs are public organisations founded on the capitalist logic [29: 19], even though they are actors involved in the production of international public goods.

#### MDBs as Socio-Economic and (Geo)Political Entities

Most MDBs share the mandate of promoting economic and social development [41]. They support marcoeconomic growth and poverty reduction, as well as sustainable development more broadly [27]. Their basic mission is to ensure funding in situations that are unattractive to the private sector [37: 105]. This means that they lend money on conditions that are less harsh than those generally imposed by commerical banks [54: 7]. MDBs do, however, raise money from the private sector to complement their activities, as well as rely on corporations to provide technical expertise and project implementation [54: 7].

But MDBs are—due to their shareholder interests—also political entities entangled in geopolitics and the tightening global competition, as all shareholder states have their own specific priorities and agendas [51: 144, 79: 238]. As put by Kearrin Sims, ‘[t]he notion that MDBs offer politically neutral financing is an illusion…’ [76:150]. In a similar manner, Engel and Baz have claimed, ‘[s]hareholder influence is a perennial issue for the MDBs’ [37]. Indeed, it is widely admitted that ‘leading shareholders exercise considerable influence over strategic directions’ [29: 108]. The rationale for establishing MDBs in the first place must also be seen as an extension of broader foreign policy goals in founding countries [39]. Hence, the World Bank has been portrayed as realising a US-led vision of development. In turn, the ADB is generally considered Japan-led [33: 125, 76: 150], and the AIIB is dominated by China [36] (see Table 1).

**Table 1:** Overview of the ADB and AIIB

	ADB	AIIB
Member states (regional members)	68 (49)	106 (47)
Total USD commitments in 2021	22.8 billion	9.93 billion
Biggest shareholder	Japan; the USA	China

Sources: [11, 12, 26]

There are both formal and informal means through which powerful shareholder states seek to impact the work of MDB. Formally, influence is embedded in the MDBs’ governance structures and in particular the voting power of shareholder member states [29: 108, 76: 150, 57]. Member states may have different views on institutional design and official policies, ranging from the appointment of staff to concrete financing decisions, thereby creating tensions that reflect in the functioning of the bank. This is evident from national strategies, which establish different priorities on how to work in specific MDBs. To illustrate, Denmark has set forth that it seeks gender mainstreaming in all aspects of the AIIB’s operations [81], yet the AIIB is not particularly known for having placed emphasis on that particular aspect of social protection. In the field of fossil fuel policies, Australia—the fifth largest shareholder of the ADB—was known to resist a total fossil fuel ban in the revised energy strategy of the bank [52]. Canberra subsequently received a lot of criticism for its failure to advance the concerns of its neighbouring states [80].

The establishment of the AIIB and ensuing politics demonstrate well how MDBs are far from apolitical entities. The USA sought to persuade its allies not to join the bank, nevertheless failing in its efforts [37: 125]. Instead, the race to join the bank included many European states and particularly the UK, whose accession to AIIB infuriated the USA [83]. The bank’s creation also raised the question of Taiwanese membership in the AIIB. The Chinese influence over the AIIB became apparent when Taiwan was rejected as a founding member of the bank [49]. Other reported examples of the political significance of MDBs claim that Japan has tried to accumulate support for its candidacy for the UN Security Council by highlighting its ADB presidency [33: 124].

#### Multi-layered Agency and Institutional Autonomy

Multilateral development banks are formed by states by virtue of a treaty and are thus international organisations under public international law. The rationale behind the creation of such institutions is functional, as the underlying thinking is that the establishment of an organisation will be a more practical and expedient way to meet the sought objectives [45]. In other words, an international organisation embodies a principal-agent relationship, in which founding states are the principal, and the organisation is the agent [58: 10]. However, states are paradoxically also part of the agent, as organisations usually have an assembly allowing for direct membership participation [58]. While there is, in the present context, no need to delve into whether the creation of an international organisation entails the transfer of sovereignty and to what degree, it is relevant to underline that member states in international organisations are crucial for the effectiveness of the organisations [28: 33]. As noted by Barros with regard to the role of member states in international financial institutions, ‘they [the member states] delineate their [the institutions’] purposes, institutional design and rules of procedure; they finance and provide material support for the operations of international organisations; they second their nationals to the latter’s organs; they affect the composition of management bodies; they control the accession procedure of new members; they participate in institutional decision-making processes; and they implement institutional decisions.’ [28: 33]. Hence, states loom behind the institutional veil.

The concept of institutional autonomy is used to discuss the degree to which an international organisation, such as an MDB, has a will of its own. Under international law, it is generally accepted that international organisations are actors both in practical and legal terms as they are taken to enjoy international legal personality. International law thus singles them out as independent actors on the international scene [28: 58], which often entails an expansion of institutional competencies beyond the original mandate. Notwithstanding the independence of MDBs to act within the field of their mandate, the role of both the lead state and the rest of the member states cannot be ignored: states use MDBs to pursue their national interests in the field of foreign policy [28: 58]. Although state control over MDBs varies, the roles of the leading state—exercising greater authority over decision-making—and the rest of the member states should not be neglected. Multilateral development banks personify multi-layered agency. They are not only independent actors in their own right, they also represent their member states, and, in particular, their lead state. If one takes the AIIB as an example, the bank represents a multi-layered reality where the interests and priorities of the bank itself are additionally instructed by China as a lead state, and the totality of AIIB members.

## The Asian Development Bank and Connectivity

### Mandate and Work

Established in 1966 with the headquarters in Manila, the Philippines, the Asian Development Bank with its 68 members is a much older MDB than the AIIB. Its long life allows for an evaluation of both how connectivity perceptions have developed within the bank and how projects aimed at augmenting connectivity have fared. ADB’s level of investments is also higher than that of its younger, regional counterpart. In 2021, its overall portfolio reached USD 118 billion, with the vast part being loans to sovereign clients, i.e. states [13: ii]. In recent years, a big portion of the portfolio consisted of investing in vaccines, due to the ongoing COVID-19 pandemic. While this activity cannot be classified as infrastructure connectivity per se, it represents so-called vaccine diplomacy or ‘vaccine connectivity’ [78].

The ADB has played a role in closing the connectivity gap in Asia [52, 70: 166], albeit it is incapable of addressing the vast needs on its own or even together with other regional development banks [30: 350]. This corresponds to its mandate, which is to ‘foster economic growth and co-operation in the region of Asia and the Far East’ [5: art. 1]. In fulfilling its mandate, the bank promotes investment, from both private and public sources, in furthering development by way of prioritising projects adding to the ‘harmonious economic growth of the region’ [5: art. 2], while taking account of existing special needs. According to its website, the bank ‘is committed to achieving a prosperous, inclusive, resilient, and sustainable Asia and the Pacific, while sustaining its efforts to eradicate extreme poverty’[7]. Other explicit priorities of the bank pertain to the coordination of development plans as well as working towards better utilisation of resources, in addition to promoting trade, particularly within Asia [7]. Infrastructure financing is not mentioned in the foundational agreement of the bank, and neither is connectivity—which is logical, given that connectivity was not a paradigm per se for pursuing development funding in the days when the bank was established. However, the ADB was reportedly involved in coining the term ‘connectivity’, when pondering responses to the 2008/2009 financial crisis together with ASEAN [43: 299].

The early attention of the bank upon domestic infrastructure has shifted to cross-border connectivity (CBC), and a concomitant broadening of how this notion is understood [62:10]. The bank’s operational priority ‘regional cooperation and integration’, has provided the framework for developing infrastructure and connectivity. This focus of the bank is dedicated to promoting ‘cross-border infrastructure, trade integration, financial links, and regional public goods’ [8]. The ADB posits that its cross-border infrastructure projects of road constructions, power lines, bridges and ports have connected members of the bank, while ‘boost[ing] growth and development througout the region’ [8]. However, connectivity has another dimension next to infrastructure projects: integration in the region has been pursued also by concluding trade agreements and creating institutions that facilitate regional integration [8].

One of the more prominent examples of ADB connectivity financing is the Mekong Region in Vietnam, which has benefitted from several connectivity projects. In 1992, the ADB established the Greater Mekong Subregion economic cooperation programme together with Cambodia, China, Laos, Myanmar, Thailand and Vietnam—an area that is home to over 300 million people. This CBC programme for a ‘natural economic area’ was afforded over USD 20 billion by the ADB in 1992–2017 [9, 65: 461]. It focused, inter alia, upon energy transmission and trade in the region, as well as inland and maritime transport and road safety, among other things [47, 65]. The Greater Mekong Subregion programme also reportedly served as an inspiration for ADB connectivity promotion in Central Asia under the so-called CAREC programme from 1997 to mid-2010s [65]. This entailed the building of six connectivity corridors among ten countries [65: 463]. Indeed, building transport corridors in economic subregions has been central to the ADB and one of its strengths in fostering connectivity [62].

The bank’s 2006 Regional Cooperation and Integration (RCI) strategy sought to ensure the increased importance of soft connectivity to balance hard infrastructure [15, 62: 10].^2^ A later evaluation of the RCI umbrella further highlighted the need for the facilitation of trade [62]. Climate change has also accelerated the call for mitigating the negative impacts of CBC, especially transport [62: 12]. This corresponds with the bank’s efforts to shift the traditional emphasis from road infrastructure to ‘multimodal’ connectivity, i.e. different modes of transport [16].

Next to the role of financing connectivity, the ADB, as well as the Asian Development Bank Institute, has also demonstrated a capacity to produce knowledge on connectivity and disseminate it. To illustrate, the ADB has developed an index to measure the level of regional cooperation and integration in Asia. This Asia–Pacific Regional Cooperation and Integration Index (ARCII) uses a number of measuring dimensions, one of which is ‘infrastructure and connectivity’ [3]. This dimension relies, among other things, on indicators like sold passenger seats on regional flights and logistics performance [4]. The ARCII shows that infrastructure and connectivity are among the main drivers for regional integration [14]. While it is difficult to assess how ARCII per se has contributed to connectivity, indexes on regional integration and their indicators help to assess the level of connectivity and meeting of concrete targets [82: 1]. For example, the ARCII demonstrates that regional integration is enhanced increasingly by connectivity that is digital [14]. Another identified role of the ADB encompasses coordination or facilitation; Menon argues that the ‘ADB has established itself as a respected facilitator and intermediary that is able to foster collaboration among a diverse set of players’ [62: 9]. Accordingly, its value for connectivity also lies in being an impartial mediator between governments, businesses, grassroot movements and other actors [62].

### Longevity in Connectivity Promotion and Influence of Japan

The ADB, along with Japan as an influential power therein, has for decades financed Asian connectivity. As Nikolay Murashkin claims, ‘Japan-led ADB was the first Asian multilateral development bank to promote connectivity within and between Asian sub-regions’ [65: 465]. The track record of advancing connectivity in Asia dates back to the post-Cold War era, when connectivity was largely a business-oriented modus operandi rather than a foreign policy tool [65: 457]. Infrastructure was understood as a substantial part of development, before the shift to securitisation with China’s introduction of the BRI. In the mid-2010s, Japan increased its financing of infrastructure in Asia by hundreds of billions. Part of this was made up of contributions to the ADB [65: 460]. Infrastructure became a central tool for Japanese statecraft, which coincided with the broader politicisation of infrastructure, as well as the Sino-Japanese rivalry in commerce [65].

The ADB has constituted one of the central funding institutions through which Japan has invested in infrastructure connectivity for a long time. As the biggest shareholder of the ADB with 15.6% of the total shares in the bank [10] (next to the USA, which has an equal share), Japanese influence in the ADB relies upon, but is not limited to, voting power. However, its substantial funding of the Asian Development Fund and staffing of the bank with high-ranking officials have also secured influence [57]. Japan is broadly viewed as being ‘the most influential regional member’ of the bank, and Japanese support is arguably needed for taking matters to the decision-making body, the Board of Directors [61].

## The Asian Infrastructure Investment Bank and Connectivity

### The Mandate and Work

The AIIB is the latest multilateral development bank to begin operation. It was launched in 2016 with 57 states as shareholders. The membership has since expanded to cover 106 states, turning the bank into an institution with veritable global ramifications [21]. The Asian Infrastructure Investment Bank is directly mandated to promote connectivity in its Articles of Agreement (AOA) [17]. Its first purpose is to ‘foster sustainable economic development, create wealth and improve *infrastructure connectivity* in Asia by investing in infrastructure and productive sectors’ [17: art. 1(1), emphasis added]. Thus, the AIIB understanding of connectivity points to it being both a means to an end and an end in itself. The preface to the AOA also opens up the connection between infrastructure, connectivity and development by contending that ‘infrastructure development expand[s] regional connectivity’ … which in turn enables economic growth, bears social development and ‘contribut[es] to global economic dynamism’ [17: preface]. The bank’s mission to further connectivity is also linked to regional cooperation and promoting economic growth [18], as connectivity is said to be ‘of critical importance’ to both efforts. The bank explains that it is filling a huge ‘connectivity gap’ between Asian economies and neighbouring states [18]. AIIB President Jin Liqun aptly frames the bank’s mission as follows: ‘[i]nfrastructure leads to connectivity with prosperity and disconnectivity from poverty’ [72: 2].

AIIB connectivity must be analysed in light of the bank’s overall principle of being ‘lean, clean and green’. These core values are said to express the bank’s desire to deliver developing states in Asia and beyond high-quality infrastructure investments based on ‘well-advised policies’ and ‘effective mechanism under sound governance’ [72: 4]. To be more precise, it entails an environmentally friendly and corruption-free AIIB that is able to decide on projects faster and more cost-effectively than other MDBs [72: 8]. This is exemplified by the bank’s approval of the first project, which only took 6 months after the start of its operations [40]. Indeed, it has been argued that the Chinese version of connectivity represents infrastructure built with lower costs, but at a quicker pace [1: 607].

Already being a recognised actor within the field of cross-border connectivity [34: 578], the AIIB aims for its connectivity projects to represent 25 to 30% of all the projects financed by the bank by 2030. In these funding decisions, priority is given to cross-border connectivity that links vital infrastructure with previously unconnected areas [19]. It is further recognised that great variation exists between different Asian regions. Other financing priorities of the bank in the field of cross-border infrastructure connectivity are projects that produce ‘measurable benefits in enhancing regional trade, investment, digital and financial integration’ [18]. The vision of increased investment in CBC is also reflected in the bank’s financing decisions, as it has over the years slowly but steadily increased its funding for cross-border connectivity projects [19].

The AIIB also sees infrastructure connectivity as broader than constructing roads or other physical infrastructure. It also relates to digital connections, whereby data and information are linked [19]. Examples of connectivity projects fulfilling the bank’s views on connectivity are, for example, the *Highway from Sylbet to Tamabil* and the *Trans Anatolian Natural Gas Pipeline Project*. While the former sought to improve traffic flows, it was also aimed at ‘eas[ing] the flow of people, goods and services between Bangladesh and India’, whereas the latter’s added value resided in connecting commerce and integrating Azerbaijan with the Southern European markets [19]. The bank does not exclude future engagement with projects related to ‘soft infrastructure’. President Jin has left the door open to new maneuvres, including projects related to social infrastructure development, namely health, education or environmental protection [72].

According to data from September 2022, the two dominant sectors of AIIB have so far been investments in the energy and transport sectors, which together amounted to more than a third of the bank’s funded commitments [22]. These included developing natural gas infrastructure, the modernisation of electricity distribution networks, road network improvement or railway electrification [19, 20]. In total, AIIB had funded 186 projects for slightly over USD 36 billion. The majority of the approved loans or investments were to sovereign states [22]. Not all, or even a majority, of these projects pertained to connectivity, however. The bank’s 2021 annual report demonstrates that both the number and the funding level of projects within the thematic priority of ‘connectivity and regional integration’ have somewhat increased in the last years: in 2019 financing in this category included four projects with a share of 14.3% of the total spending in comparison to eight projects worth of 24.2% in 2021 [26].

### Connectivity Contextualised: the China Factor and BRI

The degree to which China influences and dominates the AIIB has been much debated [56, 76, 87]. The country’s leverage is naturally relevant to the way connectivity is perceived in the policies and operations of the bank. Undoubtedly, the AIIB is a tool of economic statecraft for China [36, 85, 86]: the bank was initiated by China; its headquarters is in Beijing; China is the biggest shareholder with 26.5% of the shares [25]; the bank has a Chinese president; and, by virtue of its voting power, China enjoys a de facto veto over several issues [74, 76]. Kearrin Sims also highlights that the gap in voting power between China and the second largest shareholder, namely India, is bigger than in any other MDB [76]. Furthermore, China’s voting power is bigger than the next five biggest vote-holders combined [76: 150]. Given these facts, it appears undisputed that China plays a leading role in the AIIB. So far, China has nevertheless not let its political interests (at least publicly) affect the bank’s operations. For example, tensions between China and India in recent times have not stopped the AIIB from financing projects in India [53]. It is also noteworthy that regional members, in general, are favoured in the bank’s founding instrument: it is laid down that they must hold 75% of the voting power in the bank. Accordingly, the more non-regional members the bank has, the less they can individually influence the decision-making [48: 4].

The China factor also hinges upon the AIIB’s significant relationship to the Belt and Road Initiative (launched as One Belt, One Road Initiative, (OBOR)), which has been called ‘[t]he Archimedean point from which to interpret recent infrastructure initiatives’ [69]. What is more, the BRI, which focuses on connectivity in Eurasia with a broader spectrum of links than just infrastructure, was to be funded through a mixture of financial mechanisms, one of which was the AIIB [55, 69]. In the beginning of the bank’s journey, it was commonly held that the AIIB was ‘the mechanism for providing financial and technical support to implement the BRI, and for realizing China’s strategic aspirations with the BRI’ [1: 605]. This is unsurprising, as Chinese President Xi Jinping himself stated in November 2014, ‘China’s inception and joint establishment of the AIIB with some countries is aimed at providing financial support for infrastructure development in countries along the “One Belt, One Road”, and to promote economic cooperation’ [1: 605, citing President Xi]. However, AIIB President Jin Liqun has somewhat sought to distance the bank from the BRI by highlighting the independent nature of the AIIB. According to Jin, ‘AIIB is not created specifically to underwrite OBOR’ [72: 7]. Still, he admits that many BRI projects will be suggested to the bank for financing, and that the bank is ‘very supportive’ of the initiative [72]. This statement by President Jin, as well the China factor more broadly, indicates that the bank’s perception of connectivity and how to enhance it is closely linked to the BRI.

While it seems largely undisputed that China has considerable influence over the bank although it chooses not to directly intervene [1: 610], scholars have started to qualify Chinese dominance of the AIIB by stressing the mixture of influences that both the member states and management staff of the bank bring to the table [34: 573]. While the intention at the time of setting up the AIIB might have been to guarantee Chinese dominance, the multilateral footprint has accentuated with three of the five vice-presidents being European and almost a quarter of the shares held by non-Asian states [34, citing AIIB President Jin]. Gregory T. Chin also claims that in its early years, the AIIB operated ‘with a noticeable degree of autonomy’ [34: 573]. This was arguably made possible by India’s resistance against the BRI and the restraint exercised by Chinese finance officials [34: 573, 87]. The determination of the AIIB’s non-Asian shareholders to coordinate their work in the bank has also been interpreted as a factor mitigating Chinese influence [34: 574]. Zhu also points out that China has promised to ‘give up its veto power, “gradually”’ [87: 655].

Another move initiated by China in the field of multilateral development funding together with six MDBs is the Multilateral Cooperation Center for Development Funding, formally established in 2020 [66]. This institution, which is said to function as a bridge between the AIIB and the BRI, was proposed at the annual BRI Forum in 2017. Hosted by the AIIB, its objective is to ‘promote high-quality infrastructure and connectivity investments’ along the Belt and Road [64]. How this newest addition to the field of infrastructure connectivity will affect the AIIB is open; it is both said to increase credibility for AIIB’s operational autonomy from China [32] and spur controversy due to its unclear role in relation to the BRI [66: 83].

## Connectivity and the Three-Dimensional Agency of MDBs: Strategic, Developmental and Financial

Connectivity is part of the operations of both the ADB and the AIIB. Often connectivity is nonetheless simply described as ‘infrastructure’ since the funded projects often, or nearly always in the AIIB, relate to that sector. Infrastructure is then the ‘input’ of the banks’, which results in better connectivity—‘the output’. Connectivity features in the official policies and priorities of both banks, as the theme is central to their operations. The broader mandate held by the ADB is also reflected in the understanding of connectivity as such, as it can also entail putting in place soft structures that enable profiting from physical, hard infrastructure. This can, among other things, mean improvements in goods clearance by modernising customs and logistics [62: 2]. It is, however, noticeable that the AIIB does not exclude that broader understandings of connectivity could develop in the long-term, for example, in the field of health.

Connectivity also has two separate dimensions in the banks’ operations, as it can relate to domestic connectivity, that is, building infrastructure within a country, as opposed to CBC seeking to improve connections between states. The latter phenomenon has been actively debated by the banks in recent years, as the COVID-19 pandemic has posed a serious challenge to its realisation [20]. The MDBs also recognise that CBC depends to a large extent on regional integration and the availability of domestic connectivity, which in turn necessitates domestic reform.

The previous sections also demonstrate a different dimension of agency from the banks’ perspectives and policies on connectivity. The banks’ connections to the leading shareholders show them to be part of state-driven grand strategies, where connectivity visions coexist and compete—be it the BRI or the Japanese vision for a Free and Open Indo-Pacific [63]. As put by Gaens, Sinkkonen and Vogt, ‘connections are not only made by actors; actors are also made by connectivity’ [44: 16].

The following section will analyse the connectivity agency of MDBs against the background of the previous sections’ discussions on the ADB and the AIIB as well relevant scholarly literature. In this pursuit, the discussion will take the conceptual underpinnings of connectivity introduced by Gaens, Sinkkonen and Vogt in their analytical framework as a starting point [44], namely (1) intentionality or active agency, (2) perpetual change and (3) ‘imagined futures’. In an effort to deepen the three elements, the present discussion is inspired by Nikolay Murashkin’s analysis on strategic, developmental and mercantile aspects of Asian connectivity [65].

First, the ADB and AIIB must be understood as strategic nodes of connectivity as they are themselves connected to larger visions or strategies developed by states or aggregations of states. Second, their role as actors of development, as established in their foundational documents, has implications for the way connectivity is advanced. Third, their importance as funders of connectivity should be highlighted, along with the banking logic of their actions. While the first role highlights MDBs as objects of being connected, the second and third functions see them as subjects, that is, ‘connectors’. All three different roles that MDBs play are related and strengthen each other, yet tensions may arise among the various dimensions of their agency. Admittedly, there are other roles played by the MDBs as well, such as knowledge producer [76] and disseminator, or that of being an ‘honest broker’ [62]. They fall, however, outside the scope of this study.

### Strategic Agency

A fundamental element of connectivity is the intention to act in a way that enhances connectivity, and even a special kind of connectivity [44]. However, what seems to be a specific characteristic of the MDBs is their function of being ‘connected’ rather than acting as mere ‘connectors’. This means that one must interpret the actorness of MDBs as a cog in the wheel, forming a part of a larger connectivity vision. As Colin Flint and Cuiping Zhu explain, the economic agency is fused with geopolitical aims and visions [42: 96]. In spite of formal institutional autonomy and clear efforts by states to avoid using MDBs as direct economic and foreign policy tools, the exploration of the ADB and the AIIB clearly demonstrates that they need to be seen as institutions with geopolitical significance taking part to a varying extent in competitive connectivity between Asian actors. Giovanni B. Andornino, for example, draws attention to the economic-security nexus of the AIIB, claiming that the creation of the AIIB was not merely motivated by economic or commercial interests, but by Chinese security concerns [1]. Indeed, MDBs do not distribute aid or offer loans only based on need; studies have shown ‘statistically significant links between donor geopolitical and commercial interests, on the one hand, and access to IFI [international financial institution] resources, on the other hand’ [57: 225].

Yet, the multilateral character of MDBs is often stressed, which serves the purpose of concealing the links between different connectivity actors, hubs and corridors. The banks play a strategic role by *depoliticising* connectivity; states possessing a key role in the MDBs are careful not to directly intervene in the banks’ operations [1: 610] and may choose to channel funds through an MDB rather than resorting to bilateral funding [35]. For example, one of the reported aims of establishing the ADB in the first place was to offer Japan a ‘more neutral channel for investment’, taking that Japan had sought to occupy large parts of the region by force during the Second World War [29: 125]. Similarly, it has been held that MDBs, such as the AIIB, have provided China with some political cushioning from the negative effects of its bilateral development lending [37: 118]. Another blunt side to the depolitisation of financing via MDBs is that the banks serve as convenient scapegoats for states in politically sensitive issues or policies, such as imposing conditions on loans or enforcing them [35].

### Developmental Agency

MDBs are developmental actors by nature, as already their name indicates. They constantly seek to instigate change, inter alia by improving connections, which simultaneously feeds into their financial and banking agency. Their official policies and operational priorities are geared towards a certain understanding of how development is achieved, which depends on ‘varying beliefs about sustainable infrastructure, sustainable development, and world order’ [34: 575]. The multilayered agency of MDBs has allowed different states in a leading role to indirectly affect the MDBs and developmental policies. For example, with China playing a key role in the AIIB, it has arguably brought infrastructure back to the heart of the development discourse [37: 114], ‘the original bread and butter for MDBs’ [37: 118]. Thus, the understanding of development advanced by developed states has been challenged. The developmental understandings embedded in each MDB also affect states’ willingness to join or cooperate with a specific bank [34: 575].

The Western MDBs have commonly been viewed as advancing the so-called Washington Consensus of development [37: 113], which the ‘Beijing Consensus’ has sought to change. Whereas the former forcused on ‘“big bang” reforms’ in macroeconomics and structural changes favouring the private sector [37: 116–117], the latter pursues ‘equitable, peaceful high-quality growth’ coupled with geostrategic elements such as power projection and self-determination [73: 4]. Susan Engel and Adrian Robert Bazbauers add the focus on infrastructure investments as an additional element of the Beijing Consensus [37: 117]. Infrastructure connectivity is premised on economic growth and the rationale that by increasing connections one achieves more and more economic benefits. Indeed, with the current so-called retroliberal agenda of MDBs, infrastructure has become essential to the development agenda, partly because of the estimations of the value of infrastructure needs, and partly because of Chinese interest in this domain [29: 217]. Kearrin Sims has argued that this retroliberal perception of development has replaced the previous paradigm of poverty reduction [76, 77]. While the former focuses on economic growth, infrastructure and foreign interests, the latter pursued mitigation of poverty and civil society engagement [76: 156]. According to Sims, the ADB and the AIIB increasingly converge on their approach to development by stressing economic growth and technocratic approaches to development lending [76: 147].

### Financial and Banking Agency

The financing function of the MDBs has ensured that they play an influential role in and for infrastructure connectivity [62: 7]. The banks act as financial intermediaries and use a variety of financial instruments ranging from loans to grants in CBC funding, injecting billions of USD into the construction of roads, electricity grids, airports or railway networks. This is hardly surprising, considering that the infrastructure investment gap in Asia is commonly estimated at USD 26 trillion by 2030 [34: 571]. In fact, this funding deficit has acquired a normative status [76: 145].

MDBs are financial institutions operating in ‘money markets’ [29: 105]. Their functioning is not only affected by the core interests of shareholder states or states that play a leading role; it is also influenced by the financial market, capitalistic logic. This corresponds well with the emphasis of Gaens, Sinkkonen and Vogt on the future-oriented creation of new and profitable connections that thrive on ‘the capitalist mode of exchange and interaction in the world’ [44]. As financial actors MDBs are concerned with loan repayment, assessment of rating agencies, ‘[c]apital adequacy ratios’ and ‘loan portfolio concentration’, to name a few [50: 487]. MDBs have to both fulfil the demands for creditworthiness imposed by the money markets and attract borrowers [29: 107]. As Christopher Humphrey notes, there is an innate tension between being an actor of both ‘development’ and ‘banking’ [50]. In fact, studies have shown the banking side overshadowing the developmental mandate. As put by Ben-Artzi concerning a number of regional banks, in general they ‘function more like banks’ than agencies of development [cited in 29: 106]. Indeed, Engel and Bazbauers have stressed the existence of a ‘banking consensus’ impacting the lending and functioning of MDBs [29: 114]. Thus, next to their strategic and developmental roles, MDBs need to be understood as banks.

The link between the financing and banking actorness and connectivity lies in infrastructure, which has traditionally formed the core of MDB activities. It is generally recognised that MDBs fund projects that are unable to attract private funding. In other words, not-so-top-notch projects are financed by MDBs, which means their activities are risky in character [29: 119, 86: 367, 62: 6], both politically and financially. Financing infrastructure connectivity involves high risks politically, as difficult issues such as the resettlement of affected populations may need to be implemented, or there might be corruption [29: 119]. Moreover, political leadership in the borrowing country may change, triggering renegotiations on loan conditions. Funding massive infrastructure projects is controversial, as infrastructure is said to be ‘violent’ due to economic and social concerns [66]. Although the AIIB, for example, has arguably been cautious not to commit itself to overly risky projects in its first operative years [1: 606], it has still financed projects categorised as ‘risky’ in Nepal. One of these involves the resettlement of ethnic minorities for the construction of hydropower [29: 125]. In addition, the funding of fossil fuel projects has spurred criticism from the civil society sector, while the shift in infrastructure parlance from billions to trillions has alarmed civil society organisations [67].

It should be noted that large capital-intensive infrastructure projects are also financially risky because the MDBs need to ensure that developing states are able to pay back their loans [34: 571, 86: 367]. Therefore, utilising MDBs for connectivity financing serves to spread the financial risks involved [1: 610, 63: 17]. With the increasing debt level of poorer countries, MDBs have staff hired to perform comprehensive risk analysis [34: 571]. Another development visible in the MDBs is a tendency to steer funding towards more creditworthy states instead of those in higher need of development. In fact, like most banks, MDBs also balance the concern of their borrowers having too much debt with the necessity of providing loans [29: 51]. One way of doing this is working together with other financiers, whether MDBs, commercial banks or private sector [67]. This mixture of actors involved in infrastructure connectivity funding has triggered concern in local communities [67], as the responsibility for delivering public goods is diluted.

## Conclusion

Worldwide attention is being paid to connectivity, which is in increasing need of theorising despite being a seemingly straightforward concept. This article has sought to contribute to discussions on one particular category of actors engaged in connectivity, namely MDBs. The ADB and the AIIB were respectively analysed for the purpose of understanding how they relate to state-driven connectivity and concomitant connectivity strategies, as well as how they translate connectivity into concrete action. While the ADB has longer experience with promoting connections and regional integration, the AIIB has an explicit mandate to increase infrastructure connectivity. The case-studies of this article also testify to the significant role played by Asia in spearheading connectivity.

MDB agency in and with respect to connectivity is varied. This article limited exploration to three main agency dimensions. First, it identified that MDBs are themselves seen as nodes of connectivity attached to their most powerful shareholders. This is understandable, as this article has illustrated, by describing MDBs as multilayered actors representing the institution itself, its whole-of-bank membership and the interests of key shareholder states. The MDBs are not only in the business of connecting infrastructure; they are part of the very connectivity fabric. As such, they are used strategically for many purposes, including depoliticising financing to infrastructure connectivity that otherwise could raise concerns in a specific region. This strategic agency of MDBs thus indirectly serves geopolitical interests.

The second identified dimension of connectivity agency is related to the MDBs’ developmental role and their desire for change. In their foundational documents and official policies on operational priorities, the banks advance a particular understanding of the development and how to best achieve it. While the overall banking landscape has changed with the rise of China, it was argued that the banks have much in common: improving infrastructure connectivity leads to more economic benefits and, therewith, social and economic development.

Admittedly, there are political and economic risks involved with funding infrastructure connectivity. While the multilateral character of MDBs can be used by states to mitigate the political costs, economic risks are also notable in infrastructure connectivity. Thus, in their third (and obvious) capacity as agents of connectivity, namely as financiers, MDBs seek to spread economic risks. Simultaneously, their banking character drives them to issue loans to clients. After all, MDBs are banks that follow the money market logic.

The current exploration of MDB connectivity agency is limited both in material and geographical scope. Further research is needed with respect to MDBs and connectivity, although it is commonly held that they attract little interest. While state-driven connectivity is increasingly analysed in academic literature, it cannot be fully understood as a phenomenon without studies on actors that are not only indirectly used for statal policies, but also contribute to the formation of connectivity independently. Exploring the role of MDBs, especially in green connectivity, would be of extra merit, given the imporance of MDBs for sustainable development.

## Data Availability

All data and materials used for this study are available in scholarly databases or publicly online.

## Footnotes

1 This means that government-sponsored credit agencies, bilateral aid agencies and domestic development banks fall outside the scope of this study.

2 Soft connectivity is taken to mean the building of trust and relationships, as well as policy coordination initiatives.
